# Supplementary material for: Identity and COVID-19 in Canada: Gender, ethnicity, and minority status
Source: PLOS Glob Public Health. 2023 May 24;3(5):e0001156. doi: 10.1371/journal.pgph.0001156 (PMC10208517; doi:10.1371/journal.pgph.0001156)
Supplement: Attachment — Submitted filename: Response to reviewers .docx [file pgph.0001156.s001.docx]

**Review Comments to the Author**

**Reviewer #1:** This manuscript reports the results of an online survey investigating the relationships between socio-demographic variables and COVID-19 symptoms. In general, the manuscript is well written, although there are grammatical errors so I encourage the authors to carefully review the text. There are several areas (outlined below) where, in its current form, I do not believe the manuscript meets the publication criteria set by PLoS Global Public Health.

1. Title. "Gender and ethnic disparities ..." does not seem to reflect the results of the study. None of the statistical models found differences in the odds of reporting COVID-19 symptoms between males and females.

RESPONSE: Thank you for the comment. We acknowledge that gender and ethnic disparities were not observed in most of the results. However, there were few instances where this was observed. For gender, we found that “Men from mixed race are significantly more likely to reports experiencing COVID-19 symptoms while this is not the case for women from mixed race. We also observe that residents of British Columbia are significantly less likely to report COVID-19 symptoms, but this is only true for men and not for women.” For ethnicity, “respondents who belonged to mixed race [aOR=2.77; CI=1.18-6.48] were more likely to report COVID-19 related symptoms compared to whites.” We have also added Figure 4 to show how the results on reported COVID-19 symptoms among respondents or at least a member of their households differ by gender.

2. The data availability statement indicates data may be available from the corresponding author upon reasonable request. This does not meet the journal requirement of data being fully available at the time of publication.

RESPONSE: The survey data cannot be shared publicly as they hold potentially attributable sensitive information regarding the participants. It would therefore be unethical to make them public and would undermine the ethical committee agreement and consent process. Data can be requested to the University of Ottawa Office of Research Ethics and Integrity by researchers who meet the criteria for access to confidential data. All other relevant data are presented within the article.

3. The methods section does not contain sufficient detail about the sampling method or target population. For instance, there is limited information about the survey per se, but nothing related to the sampling methdology such as eligibility criteria, how the survey was advertised to potential participants or the sampling frame. There is a sentence in the 'strengths and limitations' section indicating that convenience and snowballing were used, but this is the only mention of the sampling methodology in the manuscript.

RESPONSE: We have provided information on the sampling method as follows “For data collection, we shared the survey in various social networks and encouraged the snowball method. We also shared the University of Ottawa's Web links via sponsored posts on Facebook in English and French via the university's institutional account. Our sampling was non-random as respondents were sampled online using convenience sampling and snowballing.”

3. There are numerous discrepancies between the information in the methods and results. Three examples are:
a) Outcome variable. From the methods "The second outcome variable was COVID-19 related symptoms among either respondent or a household member." The reported results were that 13% of respondents has at least one COVID-19 symptom, while 7% reported they or at least one household member had symptoms. Since the second outcome includes symptoms in the repondent or any household member I do not understand how the prevalence can be lower than that for the respondents. Therefore I cannot make sense of any results reported for the second outcome.

RESPONSE: Thank you for this great observation. We mistakenly presented results for experience of symptoms among household members only instead of respondent or any household member. We have now corrected that and presented the right results.

b) Gener-specific models. The methods indicate that Model 3 was the basis for the gender-specific models. This model contained all variables with the exception of number of household members (according to the results in Tables 3 and 4). However, Figure 3 which describes the results of the gender-specific models includes household size.

RESPONSE: Thank you for this comment. Figure 3 and now Figure 4 were generated from the Final model (Model 4) and not Model 3. We have corrected this in the methods section.

c) The age groups mentioned in the methods are not the same as those presented in the results.

RESPONSE: We have corrected this error in the methods section

4. Data were collected for race and minority group, and both were included in the statistical models. There seems to be potential for these two variables to be related. Have the authors assessed whether these variables show enough independence from each other to both be included in the models (eg, do the 351 "yes" responses for minority group come from specific races, rather than across all races)?

RESPONSE: Thank you for this comment. Race and minority group were independent of each other and did not show any multicollinearity.

5. Table 4. Several entries in Table 4 are incorrect. Model 3 aOR for >$100,000 is outside the 95% CI; Model 4 aOR for number of household members do not look correct in relation to the 95% CI

RESPONSE: Thank you for this great observation. We mistakenly presented results for experience of symptoms among household members only instead of respondent or any household member. We have now corrected that and presented the right results.

6. Conclusion. In the conclusion older individuals and individuals in larger households are used as examples of vulnerable populations that should be the focus of COVID-19 mitigation strategies. However, the results from this study do not support these as vulnerable populations. Older individuals had lower odds of COVID-19 symptoms, while the odds of symptoms at an individual level was not related to household size. It was only when the outcome was symptoms in respondent or >=1 household member that the size of the household become significant which is not surprising since the probability of at least one member having symptoms should increase with the number of people at risk.
RESPONSE: We have corrected the conclusion per the revised results

The authors may also like to consider the following suggestions:
7. Given the non-specific nature of many COVID-19 symptoms it is interesting that the authors have chosen to use presence of a single symptom, rather than the WHO case definition which requires fever AND cough, or at least 3 symptoms. How sensitive are your results to the definition of COVID-19 symptoms? Would the results change if fever AND cough were required, rather than a single symptom? At the moment the study could relate to any infectious disease having fever or cough as a symptom, rather than COVID-19 specifically.

RESPONSE: As shown in the methods section, our outcome variable was a composite of having fever, dry cough, shortness of breath, decreased sense of smell/taste, and other flu-like symptoms and not only fever or cough.

8. It is not clear to me why the results of Models 1 to 3 need to be presented. It appears the conclusions are drawn from Model 4, and there are no real differences in aOR as additional variables are added to the models.

RESPONSE: We thank you for the suggestion. We agree the odds do not change significantly across models. However, we consider presenting results for model 1-3 as appropriate to show the number of models we considered in this study.

9. It would be helpful if Table 1 included the actual frequencies, along with the weighted frequencies. It is mentioned that the sample was not representative, but there is no corresponding information about which groups are over-sampled or under-sampled.

RESPONSE: We have included the unweighted frequencies

10. The income variable is personal income. It is not clear how useful this information is for the household outcome since the respondent may have very different characteristics to other members of the household. In the discussion it is concluded that lower incomes means people are more exposed due to the nature of lower-paying jobs. This is an over-generalisation when you don't know the circumstances of the household. For instance, there is a big difference if the respondent income is the only income for the household versus if the respondent is not the main income-earner in the household.

RESPONSE: We included income because both outcomes include the respondent. Hence, the income is useful in understanding the outcome that includes the household

**Reviewer #2:** Overall:
The findings are potentially of interest, but given the fact that similar studies have already been published and the results widely reported, additional comparison is needed to provide sufficient justification of these results as a standalone publication.

I would recommend adding a comparison to other sources and previous publications (as referenced in the introduction). Are these results aligned with or in contradiction to these other sources and findings? How do these results compare to other countries? These should be included not only in the tables, but in charts to more clearly communicate the results.

RESPONSE: Thank you for the suggestion. We compared our results to other studies in the discussion section. Since we do not have data from other countries, we are unable to present results for other countries but only compare our findings to that of other studies as we have done in the discussion.

In addition, the authors mention the fact that data were collected for subsequent waves; I would suggest including those data here to expand the time scale and basis for analysis.
RESPONSE: Thank you for the suggestion. It is true data were collected for three waves. However, those data do not contain information that can be used for this study as they are on different issues. The variables considered in this study were only from the first wave. Hence, it will not be feasible to include that in this paper.

Specific comments:
Page 6: Missing word in first sentence, second paragraph.
RESPONSE: Missing word replaced

Materials and Methods: How was the survey disseminated? It is clear that it was a convenience sample, but where was it advertised and how were people encouraged to participate? Please provide additional information about recruitment for the convenience sample.
RESPONSE: We have provided information on the sampling and dissemination of the survey

The charts in the figures need to include units on the y-axis and appear to be lacking both figure titles and legends.

RESPONSE: We have provided the units for the y-axis and legends

**Reviewer #3**: This manuscript investigated the association between socio-demographic factors (such as gender, age, province, race, minority groups, and level of education) and COVID-19 related symptoms in Canada. An online survey was conducted to collect the socio-demographic data. This study suggested that mitigation strategies should focus on the vulnerable populations. The structure of this manuscript is clear, and the writing is well. However, this paper needs further improvements.

My comments are:

(1) Many studies have thoroughly investigated the impact of socio-demographic factors and other factors on the spreading of COVID-19 in various areas. They have confirmed that the scale of COVID-19 pandemics is associated with socio-demographic factors. Published papers concluded that vulnerable populations need more attention during the COVID-19 pandemics. The results and conclusions of this manuscript provided limited additional information on the impact of socio-demographic factors on COVID-19 pandemics. The necessity of this manuscript should be highlighted. Please highlight the necessity and innovation in the abstract and background sections.
RESPONSE: Thank you. We have now highlighted the necessity of the study. It reads: “This study is necessary because it illuminates the demographic nuances peculiar to the Canadian population during times of pandemics such as in the case of COVID-19. Also, as new strains of the COVID-19 emerge, it is important to understand the demographic disparities to be able to initiate policies and interventions that target and prioritize the most at risk sub-populations.”

(2) Materials and Methods: The authors used data from the first wave collected between July and October 2020. However, the infectious capacities of different SARS-CoV-2 variants and the symptom caused by SARS-CoV-2 variants were distinct. I suspect that the results of this paper cannot be used to guide the future prevention and control of the COVID-19 pandemics.
RESPONSE: Thank you for the comment. We consider the results as valuable because data was collected at a time COVID-19 was spreading in Canada. We agree that the infectious capacities of different SARS-CoV-2 variants and the symptom caused by SARS-CoV-2 variants were distinct. However, our data was collected in variants they were in existence at the time of the data collection

(3) Page 5, Exposure variables: please explain why you chose these eight exposure variables and give the references.
RESPONSE: We have provided references for the selection of the variables

(4) How did you deal with the potential collinearity problem among independent variables?
RESPONSE: We tested for multicollinearity but there was no evidence of collinearity.

(5) Results: I recommend the authors state the necessity and the logical role of the subsection at the beginning of each subsection.
RESPONSE: We have stated the necessity and the logical role of the subsection at the beginning of each subsection

(6) Discussion: it is better to discuss how can the results and conclusions of this manuscript illuminate the mitigation ways of the pandemics caused by SARS-CoV-2 variants of concern, such as the Omicron variants of concern.

RESPONSE: Thank you. We have now respondent by stating that: “Essentially, the findings indicate that some sub-populations including individuals from low-income earners, older adults, those of the mixed race, and those who lived in “other” provinces were at high risk of reporting COVID-19 symptoms. This implies that to resolve the mitigation ways of the pandemics, it is imperative to strengthen current preventive interventions in the identified sub-populations.”

Reviewer #4:

1. The manuscript is technically sound and well written and meets the publication criteria for PLOS Public Health. Methodology applied to answer the research question was appropriate.

RESPONSE: Thank you

2. Statistical analysis was done appropriately. The authors made sure to explain how they arrived to the final model that was used in logistic regression, to make it easier to follow, perhaps the authors should report only those results from the final statistical model. Since the paper is not about comparing statistical models, this should not be the focus of the results section.

RESPONSE: We thank you for the suggestion. We agree the odds do not change significantly across models. However, we consider presenting results for model 1-3 as appropriate to show the number of models we considered in this study.

3. The authors mentioned that they will make the data available upon request.

RESPONSE: Thank you

4. The manuscript is well written, save for very minor issues in commas and semi-colons in the introduction section and consistency of using abbreviations versus full name (e.g. WHO and World Health Organization)

RESPONSE: We have corrected these errors
