# Supplementary material for: Identity and COVID-19 in Canada: Gender, ethnicity, and minority status
Source: PLOS Glob Public Health. 2023 May 24;3(5):e0001156. doi: 10.1371/journal.pgph.0001156 (PMC10208517; doi:10.1371/journal.pgph.0001156)
Supplement: Attachment — Submitted filename: Response.docx [file pgph.0001156.s002.docx]

**Results**
A few things need to be done in the results section. Currently the tables are not easy to read.

Table 2: Are the values in the YES column in percentages, if so it needs to be clear in the table.

RESPONSE: Thank you for this. The values are in percentages. We have added the percentage sign to show them.

Tables 3 & 4: The authors did a good job with regression analysis running different models. Which model did you decide to use for your results and why? Did you do a model fit and which type did you use to assess. To make the tables easier to read, please present only the results from the model with the best fit and give a brief statement of the process.
RESPONSE: Many thanks for the suggestion. Our results were based on the final model in each table, which includes all the explanatory variables. Model fitness was checked using Pseudo R-squared goodness of fit test, with the highest value indicating the best fit model. In terms of the suggestion to present only results for the best fit model in the Tables, we think presenting the results for all the models will make it easier for readers to understand how the modelling was done and the variations across models and why our results were based on the those in the last model.

Discussion
The most important or main results are best presented in the first paragraph in 3-4 lines at most, to give readers a quick idea of what is to follow.

RESPONSE: We have re-written the first paragraph, taking this suggestion into consideration.
