# Supplementary material for: Identity and COVID-19 in Canada: Gender, ethnicity, and minority status
Source: PLOS Glob Public Health. 2023 May 24;3(5):e0001156. doi: 10.1371/journal.pgph.0001156 (PMC10208517; doi:10.1371/journal.pgph.0001156)
Supplement: Attachment — Submitted filename: Response to reviewers.docx [file pgph.0001156.s003.docx]

PGPH-D-22-00694R2
Identity and COVID-19 related symptoms in Canada: Gender, ethnicity, and minority status
PLOS Global Public Health

Dear Dr. Yaya,

Thank you for submitting your manuscript to PLOS Global Public Health. After careful consideration, we feel that it has merit but does not fully meet PLOS Global Public Health’s publication criteria as it currently stands. Therefore, we invite you to submit a revised version of the manuscript that addresses the points raised during the review process.

Please include the following items when submitting your revised manuscript:

- A rebuttal letter that responds to each point raised by the editor and reviewer(s). You should upload this letter as a separate file labeled 'Response to Reviewers'.
- A marked-up copy of your manuscript that highlights changes made to the original version. You should upload this as a separate file labeled 'Revised Manuscript with Track Changes'.
- An unmarked version of your revised paper without tracked changes. You should upload this as a separate file labeled 'Manuscript'.

Guidelines for resubmitting your figure files are available below the reviewer comments at the end of this letter.

We look forward to receiving your revised manuscript.

Kind regards,

Daniel Kim, M.D., Dr.P.H.
Academic Editor
PLOS Global Public Health

Journal Requirements:

1. Please review your reference list to ensure that it is complete and correct. If you have cited papers that have been retracted, please include the rationale for doing so in the manuscript text, or remove these references and replace them with relevant current references. Any changes to the reference list should be mentioned in the rebuttal letter that accompanies your revised manuscript. If you need to cite a retracted article, indicate the article’s retracted status in the References list and also include a citation and full reference for the retraction notice.

RESPONSE: We have ensured that all references cited are part of the reference list and the correct referencing style has been used.

2. Your manuscript is missing the following sections: Introduction. Please ensure these are present, and in the correct order, and that any references to subheadings in your main text are correct. An outline of the required sections can be consulted in our submission guidelines here:

<https://journals.plos.org/globalpublichealth/s/submission-guidelines#loc-parts-of-a-submission>

RESPONSE:

Additional Editor Comments (if provided):

For Figures 1 and 2, please use better resolution graphs and also display 95% confidence intervals for the prevalence estimates.
RESPONSE: We have revised Figures 1 and 2 accordingly.
